# Parasite load evaluation by qPCR and blood culture in Chagas disease and HIV co-infected patients under antiretroviral therapy

**DOI:** 10.1371/journal.pntd.0010317

**Published:** 2022-03-30

**Authors:** Gláucia Elisete Barbosa Marcon, Juliana de Jesus Guimarães Ferreira, Eros Antonio de Almeida, Adriane Maira Delicio, Mariane Barroso Pereira, Jamiro da Silva Wanderley, Luiz Cláudio Martins, Paula Durante Andrade, Rodrigo Gonçalves de Lima, Sandra Cecília Botelho Costa

**Affiliations:** 1 Fundação Oswaldo Cruz; Campo Grande, Mato Grosso do Sul, Brazil; 2 Laboratório de Diagnóstico de Doenças Infecciosas por Técnicas de Biologia Molecular, Faculdade de Ciências Médicas, Universidade Estadual de Campinas, Campinas, São Paulo, Brazil; 3 Departamento de Clínica Médica, Faculdade de Ciências Médicas, Universidade Estadual de Campinas, Campinas, São Paulo, Brazil; Instituto de Ciências Biológicas, Universidade Federal de Minas Gerais, BRAZIL

## Abstract

Chagas disease also known as American trypanosomiasis, is caused by *Trypanosoma cruzi* and transmitted by triatominae-contaminated feces. It is considered a neglected tropical disease that affects 6 to 7 million people worldwide. The reactivation of Chagas disease occurs when the chronically infected hosts are not able to control *T*. *cruzi* infection, generating recurrence of the acute phase. HIV is the main immunosuppressive infection that can lead to the reactivation of chronic Chagas disease in AIDS conditions. In co-infected patients, the reactivation of Chagas disease is related to their high parasite load, high HIV viral load, and CD4 T-cell counting less than 200/mm^3^, which may evolve to meningoencephalitis and myocarditis. Eight *T*. *cruzi*/HIV co-infected patients under antiretroviral therapy (ART) and ten Chagas disease patients without HIV infection that attended at Study Group of Chagas Disease, *Hospital de Clínicas*, University of Campinas (GEdoCh/HC/UNICAMP-SP) and Pontifical Catholic University of Campinas SP (PUCC/SP) were evaluated. Tests for Chagas disease were performed, such as qPCR and *T*. *cruzi* blood culture. The patient’s medical records were analyzed to verify clinical and epidemiological data, viral load, and CD4 T-cell counting since the outset of ART. For both groups, we found no statically significant differences between parasite load via blood culture and qPCR. In *T*. *cruzi*/HIV co-infected subjects, we observed a significant increase of CD4 T-cells counting and viral load decrease, which became undetectable over the years after ART. Parasites isolated from the patient’s blood culture were genotyped, being the majority of them infected with TcII and one case of mixed infection (TcII and TcV/TcVI). These results were expected according to the region of origin of the patients. We suggest that the parasite load be monitored through qPCR in *T*.*cruzi*/HIV co-infected patients. We conclude that ART in people living with HIV improves infection and immunosuppression control, enabling the natural evolution of the American trypanosomiasis.

## Author summary

Due to immunosuppression caused by HIV, Chagas disease may behave as an opportunistic infection, being an AIDS marker. When Chagas disease reactivation takes place, the increase of parasite load with disease reagudization and clinical outcomes occurs, such as meningoencephalitis and myocarditis, several times with an unpleasant prognosis. CD4 T-cells dosage and viral load of HIV may provide pieces of evidence about the immunological response against opportunistic pathogens, as well as the parasitological tests that identify *T*. *cruzi* in the blood and biological samples that give parameters for the treatment options. Via quantitative molecular method (qPCR) and parasitological method (blood culture), we verified that the co-infected patients treated with ART had a low parasite load. Chagas disease patients with no HIV had the same pattern of parasite load and they were able to control viral replication and CD T-cells maintenance, besides promoting the response capacity to *T*. *cruzi* parasite load. The ART maintenance, the serological testing for Chagas disease in HIV-positive patients, and the use of molecular diagnostic tests are significant actions for co-infected *T*. *cruzi*/HIV patients.

## 1. Introduction

Chagas disease is caused by *Trypanosoma cruzi* and transmitted by Triatominae-contaminated feces. The parasites penetrate the insect bite or contaminate the native fruits of tropical regions. These infected fruits may be the source of the Chagas disease oral infection. Also, the literature describes some other routes of transmission, such as pregnant women to newborns, laboratory accidents, and blood transfusion. American trypanosomiasis is considered a neglected disease that affects six to seven million people worldwide, mainly in developing countries [1]. As the disease has migrate from Latin-Americans to non-endemic countries, there is a concern to diagnose, to control, and to treat Chagas disease. American trypanosomiasis is characterized by an acute phase with a high parasite load that naturally evolves into the chronic phase. In this phase, parasite load is low and is generally diagnosed by serological tests. The chronic phase may present as undetermined clinical form since no clinical signs are found in the patient, or it may evolve to the cardiac and/or digestive form of the disease [2]. Based on molecular markers, seven *T*. *cruzi* lineages are described, denominated DTU (discrete typing units): TcI—TcVI and Tcbat. The strains TcII, TcV and TcVI are related to cardiac and digestive forms of the disease, being TcII prevalent in the Central Region and the Southeast of South America [3]. Chagas disease reactivation may occur when immunosuppressive medications are used in receptors, the treatment of neoplasm and other diseases, and cases of immunodeficiency, such as the co-infected individuals with Chagas disease and HIV (human immunodeficiency virus) [4]. This co-infection is characterized by high parasite load, which is detected by xenodiagnosis, blood culture, direct detection of the parasite in the blood, amplification of parasite DNA through polymerase chain reaction (PCR), and central nervous system impairment, which is a bad prognosis [5].

Reactivation of Chagas disease can be characterized as the recurrence of the acute phase that occurs when the immune system of a chronically infected host is not able to combat the infection, resulting in the reduced capability of the host to control the *T*. *cruzi* infection. The main immunosuppressive infection that can lead to the reactivation of chronic Chagas disease is HIV, and this situation has been considered a marker for AIDS diagnostic [6]. In these cases, the reactivation of *T*. *cruzi* is demonstrated by myocarditis and meninfoencephalitis. Reactivation of *T*. *cruzi* infection is related to high parasite load and CD4 T-cells counting less than 200/mm^3^. The microscopical examination of blood samples, xenodiagnosis, blood culture, and molecular tests were used to detect the reactivation of Chagas disease [7]. Molecular tests, such as qPCR, can identify Chagas/HIV co-infected patients with reactivation of the disease [8,9]. The drugs used to treat Chagas disease reactivation are benznidazole and nifurtimox [6]. HIV was isolated in 1983 and since 1996, antiretroviral therapy (ART) has been used. The current protocols indicate the ART for people living with HIV (PLHIV), regardless of the CD4 T-cells counting, and reinforce the test and the treatment strategies [10]. ART is used to prevent HIV transmission via pre-exposure prophylaxis (PrEP) and post-exposure prophylaxis (PEP) considering the following situations: HIV serodifferent couples, men who have sex with men, sex professionals, and transsexual populations. The therapy provides benefits such as reduction of morbimortality, transmissibility, and viral suppression, which is a protector factor from opportunistic infections. *T*. *cruzi* behaves as an opportunistic microorganism in co-infected individuals with HIV. Approximately, 16,100 cases of co-infection were registered in Brazil in 2018 [11]. In this study performed from 2017 to 2019, eight Chagas/HIV co-infected patients under monitoring and ART were evaluated, in addition to 10 Chagas disease patients with no HIV infection. This study evaluates the laboratory parameters related to Chagas disease parasite load, such as qPCR and blood culture, and assesses the PLHIV immune markers of CD4 T-cells and viral load to verify the possibility of Chagas disease reactivation in co-infected individuals.

## 2. Patients and methods

### 2.1 Ethics statement

This study was approved by the Research Ethics Committee of the University of Campinas, number 2,185,608. Each patient read and signed the Informed Consent Form.

### 2.2 Patients and samples

Eight *T*. *cruzi*/HIV co-infected patients were evaluated, being five treated at the Study group of Chagas Disease, *Hospital de Clínicas*, University of Campinas (GEdoCh/HC/UNICAMP); and three, treated at the Pontifical Catholic University of Campinas SP (PUCC/SP). Ten Chagas disease individuals without HIV infection were considered as the control group.

In total, 30mL of blood sample was collected in EDTA tubes from each individual. Blood and DNA samples will be stored for five years at the Biorepository of *Laboratório de Diagnóstico de Doenças Infecciosas por Técnicas de Biologia Molecular* of School of Medical Sciences of UNICAMP.

For each patient, Chagas disease was diagnosed by two serological tests performed at the *Laboratório de Patologia of Hospital de Clínicas*/UNICAMP or PUCC Campinas-SP. The diagnostic tests (Enzyme-Linked Immunosorbent Assay (ELISA), Complement Fixation Reactions (CFR), Chemiluminescence ELISA (CE), Indirect Immunofluorescence (IIF), Indirect Haemmaglutination (IH) were performed on different dates and analyzed through medical records. One patient (ChH 05) presented inconclusive serology (ELISA reagent/IIF no reagent) and they laboratory diagnosis was confirmed by standard PCR.

The medical charts were evaluated to verify the epidemiology, as well as the complementary tests to define the Chagas disease clinical form, and the exams related to HIV infection (diagnostic date, viral load, CD4 T-cell counting, and the ART therapy used).

The following data were assessed: sex, age, origin, time of Chagas disease diagnosis and HIV diagnosis, minimum CD4 T-cell count, opportunistic infections, use of ART, and Chagas disease treatment. The clinical form of chronic Chagas disease was classified as undetermined, cardiac, and/or digestive chronic disease, according to clinical, radiological, and electrocardiographic findings [4].

### 2.3 Blood culture

For each patient, 24 mL of blood was separated into four tubes and underwent blood culture. The blood was centrifuged to 1,903 x g for 10 min at 4°C. The plasma was removed and the erythrocyte pellet was washed with liver infusion tryptose (LIT) culture medium containing 10% of bovine fetal serum and 10% of penicillin/streptomycin. The pellet was resuspended in 6 mL of LIT followed by incubation at 28°C and then mixed gently twice a week. The cultures were examined microscopically (400 x magnification) and examined every 15 days for up to 120 days Some modifications were made to the original protocol, such as blood volume, homogenization interval and culture analysis, as described above [12,13]. The parasite load was considered high when three or four tubes were positive; medium, two tubes; and low, one tube.

### 2.4 *T*. *cruzi* genotyping

The parasites isolated from positive blood cultures were genotyped in DTUs [14,15]. DNA samples extracted by phenol/chloroform method were screened using a polymerase chain reaction-restriction fragment length polymorphism (PCR-RFLP) test to examine the sequence of three target loci in conjunction with their respective restriction enzymes: heat shock protein 60 (HSP60) was digested with *Eco*RV; histone H1, with *Aat*II; and glucose-6-phosphate isomerase (GPI), with *Hha*I. Then, the product sizes of each locus and digestions were analyzed in agarose gels and visualized with ultraviolet light.

### 2.5 Quantitative PCR (qPCR)

DNA isolation was performed with approximately 06 mL of total blood and underwent buffy coat isolation using 12 mL of red cell lysis buffer (0.0114 M NH_4_Cl and 0.01 M NH_4_HCO_3_) followed by centrifugation. The pellet was resuspended in 5 mL of TKM1 buffer (10 mm Tris-HCl pH 7.6, 10 mM KCl, 10 mM MgCl_2_, and 20 mM EDTA) and 1 mL of Triton X-100, and washed with TKM1, both followed by centrifugation. Each centrifugation step was performed at 8,000 x g for 5 min at 4°C. DNA extraction from buffy coat was performed using the High Pure PCR Template Preparation (Roche) kit, according to the manufacturer’s recommendations, followed by elution in 200 μL of elution buffer. The DNA samples were stored at −20°C.

**Fig 1:**
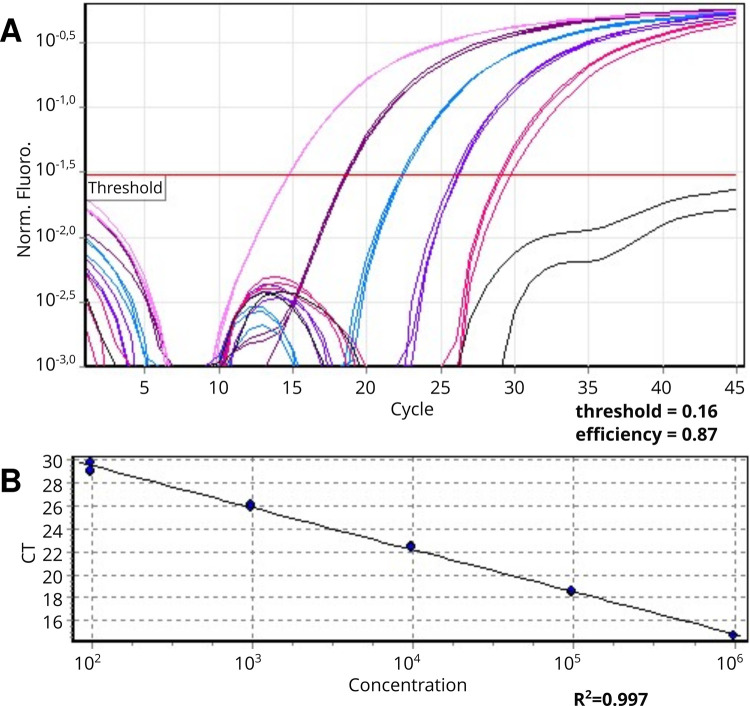
Standardization of qPCR using the *TaqMan* system. **A.** Standard amplification curve generated by 5 serial fold dilutions of DNA from blood spiked with *T*. *cruzi* epimastigotes DNA (from 9.8 x 10^5^ to 9.8 x 10^1^ parasite equivalent/mL) with threshold = 0.16, efficiency = 0.87. **B**. Linear regression curve and regression coefficient, R^2^ = 0.997.

Parasite load of the biological samples was quantified by qPCR assays targeting *T*. *cruzi* nuclear satellite with primers cruzi 1, cruzi 2, and cruzi 3 and the sequences were used as probe [16]. To build the standard curve for absolute quantification, *T*. *cruzi* obtained from an individual with Chagas disease was cultured in LIT medium to obtain 9.8 x 10^6^ Par. Eq/mL (parasite equivalent per milliliters of blood). Blood from a healthy donor was spiked with 200 μL of cultured *T*. *cruzi* and subsequent DNA extraction was performed. The DNA obtained was five-fold diluted, generating a standard curve ranging from 9.8 x 10^5^ to 9.8 x 10^1^ Par. Eq/mL. Each concentration was tested in duplicate. The dilution 9.8 x 10^1^ was considered the limit of quantification of qPCR in this study. The parameters obtained in standard curve were threshold = 0.16, efficiency = 0.87, and linear regression coefficient, R^2^ = 0.997 (Fig 1). The samples considered detectable, but not quantifiable, were those with amplification signals ≥0.1 parasite equivalents/mL. Samples presenting no amplification signal or value inferior to 0.1 Par. Eq/mL were considered not detectable. Samples without DNA (no template control) were included in each run to verify carryover contamination during sample preparation. Each reaction consisted of 1x *Taqman* Universal Master Mix with UNG (Applied Biosystems), RNase P 0.1 x (Applied Biosystems), 500 nM of each primer (Cruzi 1 and Cruzi 2), 200 nM of probe (Cruzi 3), 3 μL of DNA and Milli-Q water to complete 30 μL. The RNAse P gene (Applied Biosystems) was used to confirm the absence of PCR inhibitors and to confirm the integrity of reaction. The cycle of reactions consisted of one cycle at 50°C for 2 min, one cycle at 95°C for 10 min, and 45 cycles at 95°C for 15 s and at 58° C for 1 min in a Rotor-Gene 6000 real-time PCR cycler (Corbett Life Science).

### 2.6 Statistical analyses

Mann-Whitney test and Fisher’s exact tests were used to verify the relationship between the parasite load of *T*. *cruzi* (Par. Eq/mL) detected by qPCR, the positivity of blood culture, and patient age. Mann-Whitney test was used for comparison of blood culture and qPCR in two groups: co-infected Chagas/HIV patients and patients with Chagas disease without HIV. The Wilcoxon test was used to verify the increase and difference in the CD4 T-cells concentration in the group of Chagas/HIV co-infected patients, with a null hypothesis: median equal to zero. In every test, a 5% significance level was used, *p*-value < 0.05 [17]. The software used was Statistical Analysis System (SAS) for Windows version 9.4.

## 3. Results

The clinical and demographic characteristics of 18 patients studied are described in Table 1 (Chagas/HIV co-infected patients) and Table 2 (Chagas disease patients, without HIV). Among the 18 participants, nine (50%) were from Minas Gerais, Southeastern Brazil. Therefore, this state has the most cases in our sample, whereas the state of São Paulo is the origin of five patients (27.7%). One patient of each of the following states was included in the study: Ceará, Bahia, Mato Grosso do Sul, and Paraná. Currently, all patients dwell in the municipality of Campinas and its metropolitan area.

**Table 1:** Chagas disease dates in co-infected patients.

Patient	Age (years)	Origin State/BR	Collection blood	CD diagnosis	Month / year CD diagnosis	Clinical features	CD treatment	Blood Culture (4 tubes)	DTUs	Par Eq/mL qPCR
ChH01	50	Ceará	06/25/18	CL ELISA: R IIF: 140	03/2013	Cardiopathy	Yes	Negative	-	0.42
ChH02	72	São Paulo	06/25/18	IIF: 1/160 ELISA: R	04/2009	Indeterminate	No	Positive (++)	II	15,500,000
ChH03	65	São Paulo	07/19/18	IH: 1/1280 ELISA: R	07/1999	Cardiopathy	No	Positive (++)	II and V/VI	0.1
ChH04	72	Minas Gerais	08/27/18	IIF: 1/1280 ELISA: R	07/2012	Indeterminate	No	Negative	-	0.06
ChH05	41	Minas Gerais	09/18/18	IIF: NR ELISA: R PCR: POS	02/2012	Indeterminate	No	Negative	-	0.08
ChH06	76	São Paulo	11/21/18	IH: 1/320 IIF: 1/1280	05/2009	Cardiopathy	No	Positive (+)	II	0.6
ChH07	76	Minas Gerais	11/21/18	Wi IIF: 1/320	08/2008	Cardiopathy	No	Negative	-	0
ChH08	46	Minas Gerais	12/03/18	ELISA: R IIF: 1/160	07/2013	Indeterminate	No	Negative	-	0.06

ChH - patient co-infected Chagas disease and HIV; BR - Brazil; P - positive; Wi - without information; CD - Chagas disease; CL ELISA - chemiluminescent enzyme-linked immunosorbent assay; R - reagent; IIF - indirect immunofluorescence reagent ≥ 1/40; IH - indirect hemagglutination; ELISA - enzyme-linked immunosorbent assay; PCR - polymerase chain reaction; DTUs: discrete typing units; Par Eq/mL: parasites equivalents per milliliters; qPCR - quantitative polymerase chain reaction.

**Table 2:** Chagas disease dates in patients without HIV.

Patient	Age (years)	Origin State/BR	Blood collection	CD Diagnosis	Month / year CD diagnosis	Clinical features	Blood Culture (4 tubes)	DTUs	Par Eq/mL qPCR
C01	67	São Paulo	09/17/18	ELISA: R IIF: 1/160	08/1996	Indeterminate	Positive (++++)	II	0.347
C02	45	Minas Gerais	09/17/18	CL ELISA: R IIF: 1/640	11/2016	Cardiopathy	Positive (+)	II	0.5
C03	51	Bahia	10/15/18	ELISA: R IIF: 1/40	03/2004	Cardiopathy	Negative	-	0
C04	50	Minas Gerais	10/15/18	CL ELISA: R IIF: 1/320	10/2017	Indeterminate	Positive (+)	II	0.239
C05	68	Minas Gerais	10/29/18	CFR: 1/32 IIF: 1/80	01/1981	Cardiopathy and Digestive form	Positive (++)	II	0.102
C06	59	Minas Gerais	10/29/18	ELISA: R IFI: 1640	05/1998	Cardiopathy and Digestive form	Positive (+++)	II	0.0275
C07	55	Mato Grosso do Sul	11/12/18	-	11/2012	Cardiopathy	Negative	-	0.047
C08	68	Paraná	11/26/18	CL ELISA: R	04/2017	Cardiopathy	Negative	-	0.057
C09	59	Minas Gerais	11/26/18	CL ELISA: R IIF: 1/1280	10/2014	Cardiopathy	Negative	-	0.032
C10	64	São Paulo	12/03/18	ELISA: R IIF: 1/80	10/1991	Indeterminate	Negative	-	0.028

C01 - patient with Chagas disease without HIV; BR - Brazil; Wi - without information; CD - Chagas disease; CL ELISA - chemiluminescent enzyme-linked immunosorbent assay; R - reagent; IIF - indirect immunofluorescence reagent ≥ 1/40; CFR- complement fixation reactions, reagent ≥ 1/32; ELISA - enzyme-linked immunosorbent assay; DTUs: discrete typing units; (+): number of positive tubes for blood culture; Par Eq/mL: parasites equivalents per milliliters; qPCR quantitative polymerase chain reaction.

The standard curve generated for parasite quantification by qPCR showed 87% of efficiency and R^2^ = 0.99 for linear coefficient, employing serial dilutions from 9.8 x 10^5^ to 9.8 x 10^1^ parasites equivalents/mL (Fig 1). The samples considered detectable were those with amplification signals ≥0.1 parasite equivalents/mL.

Considering *T*. *cruzi* parasite load evaluated by blood culture, eight out of 18 patients were positive (8/18, 44%), and 44% (8/18) of patients presented qPCR results detectable (Tables 1 and 2). We found no statistical significance related to age (*p* = 0.42), qPCR (*p* = 0.22), and blood culture (*p* = 0.66) when compared to the two studied groups, Chagas/HIV co-infected patients and Chagas disease individuals without HIV (Mann-Whitney test, Fisher’s exact test, confidence interval *p* ˃ 0.05) (Table 3). No statistical significance was found when comparing the parasite load obtained by qPCR and blood culture in the two evaluated groups, whereas the Chagas/HIV co-infected subjects presented *p* = 0.072 and Chagas disease patients without HIV presented *p* = 0.075, based on the Mann-Whitney test, *p* ˃ 0.05 (Table 4).

**Table 3:** Statistical analysis—Co-infected Chagas HIV and patients without HIV.

Characteristics		Co-infected Chagas/HIV (n = 8)	Chagas without HIV (n = 10)	Total (n = 18)	P value
Sex	male female	5	5	10	-
		3	5	8	
Age	range mean median	41–76	45–68	41–76	0.42^1^
		62.25 ± 14.35	58.60 ± 8.21	60.22 ± 11.13	
		68.5	59	61.5	
*T*. *cruzi* Par Eq/mL	range mean median	0.0–15,000,000	0.0–0.5	0.0–15,000,000	0.22^1^
		1,875,000.16 ± 5,303,300.79	0.13 ± 0.16	833,333.48 ± 3,535,533.87	
		0.09	0.05	0.07	
Blood culture	positive negative	3 (37.5%)	5 (50%)	8 (44.4%)	0.66^3^
		5 (62.5%)	5 (50%)	10 (55.6%)	

^1^ based on the Mann-Whitney test/^3^ based on Fisher’s exact test; CI = Confidence Interval, P ˃0.05.

**Table 4:** Comparison between blood culture and qPCR.

Group	Blood culture (n)	qPCR				P value^1^
		range	average	median	standard deviation	
**Co-infected Chagas/HIV**	Negative (5)	0–0.4	0.12	0.06	0.06	0.072
	Positive (3)	0.1–15,000,000	5,000,000	0.6	8,660,254	
**Chagas without HIV**	Negative (5)	0–0.05	0.03	0.03	0.02	0,075
	Positive (5)	0.02–0.5	0.22	0.20	0.19	

^1^based on the Mann-Whitney test; CI = Confidence Interval, P ˃ 0.05.

In Chagas/HIV co-infected group, the parasite load evaluated by blood culture indicated only three patients with positive tubes (ChH02, ChH03, and ChH06), considered with medium and low parasitemia. Via qPCR, the *T*. *cruzi* was not detected in four of eight analyzed samples, in accordance with the negative results of four patients evaluated by blood culture (ChH04, ChH05, ChH07, and ChH08). The patient ChH01 diverged, showing negative blood culture and qPCR detectable. Low parasite load in Chagas disease patients not co-infected was verified by qPCR and 50% of positive blood culture (5/10). In this group, the results of two assays were concordant in nine of 10 patients tested (90%), being qPCR detectable and positive blood culture in four patients (C01, C02, C04, and C05); qPCR not detectable and negative blood culture in five patients (C03, C07, C08, C09, and C10); and only one patient was discordant, with qPCR undetectable and positive blood culture (C06) (Table 2). The increase of CD4 T-cells during ART was statistically significant for Chagas/HIV not co-infected patients, with *p* = 0.03 (Wilcoxon test, *p* < 0.05) (Table 5). The average increase of CD4 T-cells number was 2.48 higher than at the beginning of the treatment.

**Table 5:** Analyses of CD4 T-cells.

CD4 T cells (cell/mm3)	Range	Average	Median	Standard deviation	P value^1^
**Start ART**	143–463	305	272	129.09	**0.03**
**Collection time**	358–1104	696	675.5	245.16	
**Difference**	177–815	391	311.5	241.33	

^1^ Based on Wilcoxon test for related samples. Null hypothesis: median equal to zero. CI = Confidence Interval, P < 0.05.

ART - antiretroviral therapy

### 3.1 Parasite load and viral markers in Chagas/HIV co-infected patients

Among the co-infected patients studied, four subjects were female and four were male. The average age was 62.25 ± 14.35 years, with a median of 68.5 years (Table 3). At the beginning of antiretroviral therapy, the average of CD4 T-cells was 454.63 ± 378.61 cells/mm^3^ and the median was 372.50 cells/mm^3^. At the time of sample collection for this study, the CD4 T-cells average was 696.00 ± 245.16 cells/mm^3^ and the median was 675.50 cells/mm^3^ (Table 5). The mean viral load (VL), at the beginning of the treatment, was 80,406.38 ± 84,837.75 copies/mL of blood and the median was 65,818.50 (133–271,772) copies/mL. After ART, the viral load was undetectable in the eight evaluated cases. The medicines used in antiretroviral therapy, the CD4 T-cells counting, and the viral load are described in Table 6.

**Table 6:** Clinical dates in coinfected Chagas/HIV patients.

Patients	HIV diagnosis (month/year)	Category HIV/AIDS	Opportunistic Infection	Ouset of ART (years of terapy)	Viral Load (copies/mL) Outset of ART	Viral load (copies/mL) collection date	CD4 T-cells (cells/mm^3^) Outset of ART	CD4 T-cells (cells/mm^3^) collection date	ART scheme
**ChH 01**	01/2013	-	*Helycobacter pilory*	10/2013 (05)	62,496	Not detectable^1^	456	711	AZT, 3TC, EFV
**ChH 02**	03/1996	A2	No	09/2003 (15)	133	Not detectable	1341	Wi	AZT, 3TC, EFV
**ChH 03**	08/2007	B3	*Candida albicans*	08/2008 (10)	69,141	Not detectable	289	1,104	AZT, 3TC, EFV, TDF, DTG
**ChH 04**	06 /2006	C3	*Herpes simplex; H*. *zoster*	2007 (11)	271,772	Not detectable	143	358	ABC, EFV, 3TC
**ChH 05**	01/2012	A2	*Blastocystis hominis* *Endolimax nana* *Entamoeba histolytica*	03/2014 (04)	8,241	Not detectable	463	640	TDF, 3TC, EFV
**ChH 06**	02/2009	B2	No	08/2010 (08)	7,5327	Not detectable	255	771	AZT, 3TC, NVP
**ChH07**	08/2008	A2	No	11/2010 (08)	49,564	Not detectable	224	592	AZT, 3TC, EFV
**ChH08**	06/2013	A2	No	09/2013 (05)	10,6577	Not detectable	466	Wi	TDF, 3TC, ATV, RTV

HIV - human immunodeficiency virus; ChH - patient co-infected Chagas disease and HIV; AIDS - acquired immunodeficiency syndrome; ART - antiretroviral therapy; AZT - zidovudine; 3TC - lamivudine; NVP - nevirapine; ABC - abacavir; EFV - efavirenz; TDF - tenofovir; ATV - atazanavir; DTG - dolutegravir; RTV - ritonavir. ^1^Not detectable - below detection limit of 50 viral particles/mL; Wi - without information.

The patients ChH01, ChH05, ChH07, and ChH08 presented negative blood culture and the *T*. *cruzi* parasite load was undetectable by qPCR assay (Table 1). The patient ChH01 received benznidazole treatment at a dosage of 5 mg/kg/day over 30 days in 2014. The ART started between 2010 and 2013. In 2018, the year this study was performed, these patients showed an undetectable viral load, in addition to the increase of CD4 T-cells rates (Table 6) compared with the beginning of the treatment (2.48 times).

The patient ChH02 aged 72 years, with the undetermined form of Chagas disease, presented 15,500,000 Par. Eq/mL of blood evaluated by qPCR, showing high parasitemia. In this case, the blood culture was positive in two out of four (2/4) tubes evaluated. The genotyping indicated the parasite belonging to the DTU TcII (Table 1). In 2003, at the beginning of antiretroviral therapy, the viral load was 133 viral copies/mL of blood, and the CD4 T-cells counting was 1,341/μL of blood. In June 2018, at the time of this study and 15 years after the onset of ART, the viral load was undetectable and the CD4 T-cell counting was not registered (Table 6).

The patient ChH03 aged 65 years, with the cardiac form of Chagas disease, showed 0.1 Par. Eq/mL of blood. The blood culture was positive in two out of four (2/4) tubes analyzed. In this patient, two *T*. *cruzi* subtypes were found: Tc II and TcV or TcVI (Table 1). In 2008, when ART began, the viral load was 289 cells/μL. He presented oral candidiasis as an opportunistic infection. After 10 years of antiretroviral monitoring, the viral load was undetectable and CD4 T-cells counting was 1,104 cells/μL (Table 6).

The patient ChH04 aged 72 years, presented an undetermined form of Chagas disease. The parasite load by qPCR was undetectable and negative blood culture, being parasite genotyping not possible (Table 1). In 2007, the viral load was quantified in 271,772 viral copies/mL of blood and the CD4 T-cells counting was 143 cells/μL. He had herpetic keratitis and *Herpes zoster*. After 11 years of ART, at the time of collection, the viral load was undetectable and the CD4 T-cells counting was 358 cells/μL (Table 6).

The patient ChH06 aged 72 years, presented the cardiac form of Chagas disease, with positive blood culture in one out of four (1/4) tubes evaluated and *T*. *cruzi* TcII. The qPCR detected 0.6 Par. Eq/mL of blood (Table 1). The ART started in 2010, and the viral load was 75,327 viral copies/mL and the CD4 T-cells counting was 255 cells/ μL. In 2018, at the time of collection, the viral load was undetectable and CD4 T-cells counting was 771 cells/μL (Table 6).

### 3.2 Parasite load in Chagas disease patients without HIV infection

In this group, parameters were evaluated concerning Chagas disease of 10 patients (C01-C10), out of which five are female and five are male. The average age was 58.60 ± 8.21, and the median was 59 years. According to the data, the maximum time of Chagas disease monitoring was 37 years, and the minimum, one year (Table 2). Among the 10 patients, five of them presented cardiac form, three presented the undetermined form, and two presented concomitant cardiac and digestive forms. Only one patient of this group (C03) received antiparasitic therapy with benznidazole dosing 5mg/Kg/day for 17 days. However, the patient had allergy to the medicine and the treatment was interrupted (Table 2).

Five patients showed positive blood cultures and genotyping classified in TcII DTU (C01, C02, C04, C05, and C06).

The patient C01 aged 67 years, showed the undetermined form of Chagas disease and had positive blood culture in all tubes evaluated (4/4), characterizing high parasite load. The quantification by qPCR was 0.3 Par. Eq/mL of blood. The patient C02 aged 45 years, with the cardiac form of Chagas disease, had positive blood culture in one out of four (1/4) analyzed tubes and the qPCR detected 0.5 Par. Eq/mL of blood.

C04 patient aged 50 years, with the undetermined form of Chagas disease, presented positive blood culture in one of four tubes evaluated (1/4), and qPCR showing 0.2 Par. Eq/mL of blood. The patient C05 aged 68 years, with the concomitant cardiac and digestive form of Chagas disease, presented positive blood culture in two out of four (2/4) analyzed tubes and qPCR detecting 0.1 Par. Eq/mL of blood. The patient C06 presented the cardiac and digestive forms of Chagas disease, with three positive tubes in the blood culture (3/4) and the qPCR showed no DNA parasite detection (Table 2).

The patients C07, C08, C09 presented the cardiac form of Chagas disease and C10, the undetermined form. Blood culture analyses were negative, and the parasite was not detected by qPCR in these four patients (Table 2).

## 4. Discussion

In the outpatient clinic for Chagas disease and HIV care, eight patients were selected showing Chagas/HIV co-infection, all of them under antiretroviral therapy. For comparative analyses, 10 individuals with Chagas disease, without HIV infection were also evaluated. Due to the limited number of samples, we found no significant statistical differences when comparing the parasite load evaluated by blood culture and qPCR. However, in the Chagas/HIV co-infected patients, we observed a significant increase of CD4 T-cells counting and a decrease of viral load to undetectable levels over the years of antiretroviral therapy.

In reports from the literature, blood culture for *T*. *cruzi* presented sensitivity ranging 38%–53% in patients with Chagas disease [18,19]. In this study, the blood culture positivity was 44% (8/18), being within the range reported by other authors. Considering the co-infected group, the positivity was 37.5% (3/8), also within the variation reported in the literature, demonstrating that the co-infection did not change the parasitemia, which is compatible with the natural evolution of chronic Chagas disease. Adding to the low sensitivity, the blood culture is laborious and can easily be contaminated by other microorganisms, besides its interpretation is subjective. However, the test is one of the tools that allow to genotype and to quantify the parasite, presenting advantages to xenodiagnosis. In this study, among the 18 patients evaluated, only two of them presented discordant blood culture results related to qPCR (C06 and ChH01). This fact can be justified by diverse factors such as parasite intermittence at the moment of the sample collection, variable parasitemia in chronically infected individuals, the complexity of the *T*. *cruzi* life cycle, low parasite load, and low sensitivity of the blood culture in the chronic phase of Chagas disease, PCR positivity also depends on DNA extraction conditions, amplification target, and primers [20].

Among the co-infected patients, ChH02 presented the undetermined form of Chagas disease. This patient received a diagnosis in 2009, did not receive specific *T*. *cruzi* treatment and did not present the clinical evolution of Chagas disease. His HIV infection is under control due to the use of ART since 2003. This patient presented high parasitemia observed by qPCR (15,500,000 Par. Eq/mL) and moderate parasitemia by blood culture (2/4 positive tubes), which could suggest reactivation of Chagas disease. However, no clinical data indicated *T*. *cruzi* reactivation, and the VL was undetectable, which is unusual in cases of Chagas disease reactivation. In the literature, it has been described that those high levels of parasitemia detected by xenodiagnosis and blood culture in co-infected patients may not define the reactivation [7]. Thus, such data may function as a possible marker, suggesting a possible risk of the disease reactivation in these patients, requiring more adequate monitoring of the clinical and parasitological condition. On the other hand, high parasitemia can be found in individuals with Chagas disease, without co-infection, although infrequent, not reactivating the disease.

*T*. *cruzi* quantification with blood culture or qPCR is influenced by variables such as age, patient’s immunological status, *T*. *cruzi* subtype, and the fact of the circulating parasites appear in an intermittent way [20,21]. The qPCR is a method that shows high sensitivity and specificity, however, when the quantification limit is calculated according to the described protocols, the samples with very low parasite load are not quantified, they are only detected [22]. A previous study considers samples with detectable parasite load, but not quantifiable when monitoring the parasite load in chronic Chagas disease patients [19]. Therefore, in this study, we considered samples with detectable results varying from 0.1 Par. Eq/mL to 15,500,000 Par. Eq/mL in co-infected patients. In Chagas disease patients without HIV infection, the parasite load varied from 0.1 to 0.5 Par. Eq/mL (Table 3). In this research, it should be considered that the volume of the blood collected was 6 mL, only once, which can affect the quantitative results. We found a sensitivity increase when two samples are collected [19] or a greater volume of samples is processed. The genotyping was performed in the eight isolated from positive blood cultures, three of Chagas/HIV co-infected group and five of Chagas disease group without HIV infection. Among the eight isolated, seven of them belong to the TcII group, and one patient (ChH03) presented two *T*. *cruzi* lineages (DTUs TcII and TcV/TcVI). It was not possible to differentiate the subgroups TcV from TcVI. The patients who have the samples genotyped were from the Southeastern Brazil (states of São Paulo and Minas Gerais), confirming the prevalence of subtype TcII [18,23]. These patients present the clinical forms undetermined, cardiac or mixed (i.e. cardiac and digestive), demonstrating no correlation between the genetic profile and clinical forms existing in the evaluated groups, corroborating with the pieces of evidence previously described [6,24,25].

The patient ChH03 (TcII and TcV/TcVI) has Chagas/HIV co-infection, was born in state of São Paulo, and has the cardiac form of Chagas disease. The TcV and TcVI subtype is clearly associated with human Chagas disease in countries of South America, regardless of the clinical manifestation, cardiac or digestive form. We reinforce that the selection of parasite subpopulation can occur in culture medium, or moreover, the selected DTU does not reflect all clonal populations from circulation or tissue due to different characteristics of the parasite [3].

Evaluated co-infected patients underwent a follow-up for HIV infection and used a combined antiretroviral therapy. We observed that at the beginning of HIV diagnostic, the viral load was elevated (average 80,406.38 ± 84,837.75, median 65,818.50) and the antiretroviral therapy administered over the successive years allowed the viral load to became undetectable, characterizing the virological suppression with VL <50 copies/mL [26].

Inversely proportional, the quantification of CD4 T-cells, one of the indicators of the immunological status, increased on average 2.48 times during the years of follow-up. Reports in the literature have demonstrated the relationship between the reactivation of the Chagas disease with a decrease in CD4 T-cells and increase in VL. Research conducted in the mid-1990s and published before the 2000s showed a reactivation profile of 13.8–20% [5,8,27]. In this study, the results of the eight co-infected evaluated by blood culture, qPCR, VL, and CD4 T-cells, showed that the patients are immunologically able to control the infections, allowing until now, the natural evolution of chronic Chagas disease. This observation was confirmed by the parasite quantification that demonstrated low levels and even undetectable *T*.*cruzi* DNA in most cases evaluated, showing absence of the Chagas disease reactivation.

The resulting data in this study verified that antiretroviral therapy allows for the control of HIV infection and an immunological status able to avoid the reactivation of the Chagas disease, corroborating results found in the literature on the follow-up of co-infected patients under ART. In this study we observed a decrease of VL and increase of CD4 T-cells levels, ensuring immunity to these patients. [26]. The smaller time of ART evaluated in this research was four years and, the longest was 15 years. The elevated parasite load of co-infected *T*. *cruzi*/HIV individuals before the effective implementation of ART and the reduction of the parasites after the therapeutic implementation, as demonstrated in this study, reinforce the establishment of immunity due to virological suppression promoted by ART.

The medical chart analyses of Chagas-HIV patients do not indicate central nervous system (CNS) impairment or recrudescence of clinical forms of Chagas disease in the studied cases. Previous studies showed Chagas disease reactivation in cases of co-infection, being the CNS impairment the main grievance and cause of death [5,28,29].

In HIV infections, ART is obligatory in cases of co-infection. The levels of laboratory abnormalities decrease when using ART for a long time. Although we did not use previous data about Chagas disease parasite load in patients of this study, the value found confirms the significance of ART and reinforces the need for quantitative tests for *T*. *cruzi* in co-infected patients at least once a year or when there are suspect of reactivation of the Chagas disease as well as direct parasitological tests in the blood and other biological fluids are recommended, mainly in liquor in cases of CNS involvement. Thus, the antiparasitic therapy may be implemented with the detection of high parasite load, decrease of CD4 T-cells, and increase of VL [11]. The application of benznidazole must take in count the cost benefit to the patient. The age must be considered, as well as comorbidity and side effects. The monitoring of these cases with qPCR may direct the intervention with antiparasitic therapy.

In this study, only one co-infected patient (ChH01) used benznidazole over 30 days in 2014. The choice of not treated cases most of the time allowed to evaluate the importance of quantitative methods used, beyond the monitoring of ART therapy. It is important to evaluate more Chagas HIV co-infected patients, treated and not treated specifically for Chagas disease.

In the last years, we observed a decrease of newly infected patients by *T*. *cruzi* due to control of the vectorial transmission in Brazil, screening in the blood banks, and the rural exodus. Serological inquiring performed for HIV in Chagas disease patients showed 1.3–5% of co-infection. We recommend that all the HIV seropositive should be evaluated regarding the presence of Chagas disease. All patients infected by HIV should underwent Chagas disease serology [30], regardless of the dwelling region in Latin America, as well as Europe, United States, and other places of the world.

In cases of Chagas/HIV co-infection, we suggest the use of sensitive, specific, and quantitative methods, such as the qPCR for follow-up of parasite load. Even with published protocols, the financing for research to standardize these diagnostic tests and studies of Chagas disease is necessary, as well as for the quantitative tests, which can genotype the *T*. *cruzi* and help the treatment follow-up. The qPCR is a current tool and can quantify the parasite and monitor the *T*. *cruzi* parasite load in cases of immunosuppression.

As a limitation of the study, we consider the low number of patients, and that the casuistry should be increased to support the results found. About the methodologies used, even with good protocols for qPCR available [31,32], a profile of the studied population, the type of equipment, and reagents available are scarce, considering that this is a neglected disease in developing countries. As an in-house technique, the parameters of quantification may vary, whereas each study should be standardized regarding the standard curve, the detection limit, and other interpretative questions about the samples [19]. For these reasons, finding a standardization concerning the molecular techniques in the chronic phase of the Chagas disease is challenging and essential.

Considering the study performed, ART in people with *T*. *cruz*i/HIV infection promotes control of viral replication and immunosuppression, preventing complications of Chagas disease, such as its reactivation. It is concluded that if the ART maintains the immune status and consequently the controlling parasitological, therefore, monitoring these cases with qPCR may direct an intervention with benznidazole.
